# Chondro/Osteoblastic and Cardiovascular Gene Modulation in Human Artery Smooth Muscle Cells That Calcify in the Presence of Phosphate and Calcitriol or Paricalcitol

**DOI:** 10.1002/jcb.22779

**Published:** 2010-07-27

**Authors:** V Shalhoub, EM Shatzen, SC Ward, J-I Young, M Boedigheimer, L Twehues, J McNinch, S Scully, B Twomey, D Baker, P Kiaei, MA Damore, Z Pan, K Haas, D Martin

**Affiliations:** 1 Department of Metabolic Disorders, Amgen, Inc.Thousand Oaks, California; 2 Department of Computational Biology, Amgen, Inc.Thousand Oaks, California; 3 Department of Molecular Sciences, Amgen, Inc.Thousand Oaks, California; 4 Department of Pathology, Amgen, Inc.Thousand Oaks, California; 5 Department of Lead Discovery, Amgen, Inc.Thousand Oaks, California

**Keywords:** cardiovascular diseases, smooth muscle, apoptosis, phosphate

## Abstract

Vitamin D sterol administration, a traditional treatment for secondary hyperparathyroidism, may increase serum calcium and phosphorus, and has been associated with increased vascular calcification (VC). In vitro studies suggest that in the presence of uremic concentrations of phosphorus, vitamin D sterols regulate gene expression associated with trans-differentiation of smooth muscle cells (SMCs) to a chondro/osteoblastic cell type. This study examined effects of vitamin D sterols on gene expression profiles associated with phosphate-enhanced human coronary artery SMC (CASMC) calcification. Cultured CASMCs were exposed to phosphate-containing differentiation medium (DM) with and without calcitriol, paricalcitol, or the calcimimetic R-568 (10^−11^–10^−7^ M) for 7 days. Calcification of CASMCs, determined using colorimetry following acid extraction, was dose dependently increased (1.6- to 1.9-fold) by vitamin D sterols + DM. In contrast, R-568 did not increase calcification. Microarray analysis demonstrated that, compared with DM, calcitriol (10^−8^ M) + DM or paricalcitol (10^−8^ M) + DM similarly and significantly (*P* < 0.05) regulated genes of various pathways including: metabolism, CYP24A1; mineralization, ENPP1; apoptosis, GIP3; osteo/chondrogenesis, OPG, TGFB2, Dkk1, BMP4, BMP6; cardiovascular, HGF, DSP1, TNC; cell cycle, MAPK13; and ion channels, SLC22A3 KCNK3. R-568 had no effect on CASMC gene expression. Thus, SMC calcification observed in response to vitamin D sterol + DM may be partially mediated through targeting mineralization, apoptotic, osteo/chondrocytic, and cardiovascular pathway genes, although some gene changes may protect against calcification. Further studies to determine precise roles of these genes in development of, or protection against VC and cardiovascular disease are required. J. Cell. Biochem. 111: 911–921, 2010. © 2010 Wiley-Liss, Inc.

Vascular calcification (VC) is widespread in chronic kidney disease-mineral and bone disorder (CKD-MBD) patients with secondary hyperparathyroidism (HPT) receiving dialysis [Moe et al., 2007], and has been associated with cardiovascular disease and mortality [Blacher et al., 2001; Raggi et al., 2002]. VC in large and medium sized vessels is a highly regulated, cell mediated, multi-factorial process [Ketteler and Giachelli, 2006] associated with a repertoire of chondro/osseous proteins [Jono et al., 2000; Tyson et al., 2003]. Therapies used to control secondary HPT, including vitamin D sterols and calcimimetics, have varying direct and indirect effects on VC [Wolisi and Moe, 2005]. Although vitamin D sterols lower serum parathyroid hormone (PTH) levels, they have been associated with serum calcium (Ca) and phosphorus (P) elevations in patients receiving dialysis [Tentori et al., 2006]. This increased serum mineral burden can increase the risk for VC and mortality [Kalantar-Zadeh et al., 2006; Moe et al., 2007]. In addition to serum Ca and P mineral imbalance [Goodman et al., 2000b], contributing factors to VC may include dysregulation of mineralization inhibitors [Ketteler et al., 2003; Lomashvili et al., 2005], apoptotic mechanisms [Reynolds et al., 2004], and current therapies such as calcium-based phosphate binders and vitamin D sterols [Goodman et al., 2000b; Wolisi and Moe, 2005]. The interrelationships between these factors, the precise timing of molecular events, and other possible factors are currently unknown.

While vitamin D is necessary for patient health and to control secondary HPT in dialysis patients, the harm of too little or too much vitamin D is becoming increasingly appreciated. In pre-clinical CKD-MBD models, pharmacological levels of the vitamin D sterol calcitriol increased VC [Haffner et al., 2005; Wu-Wong et al., 2006; Cardus et al., 2007], but it was not assessed whether this was a result of direct effects on vascular smooth muscle cell (SMC) in the setting of deranged minerals, indirect via increased mineral levels, or both. Early reports of in vitro model systems demonstrated that bovine and human VSMC spontaneously converted into chondro/osseous phenotypes that calcified [Bostrom et al., 1993; Watson et al., 1994; Shanahan and Weissberg, 1999; Tyson et al., 2003]. Calcification was accelerated by phosphate [Shioi et al., 1995; Jono et al., 2000; Reynolds et al., 2004] and more-so by phosphate + dexamethasone [Mori et al., 1999] or vitamin D [Jono et al., 1998]. However, subsequent reports on direct effects of vitamin D sterols on SMC calcification were conflicting. We reported that vitamin D sterols (calcitriol and paricalcitol) increased phosphate-induced calcification and chondro/osteoblastic gene expression [Shalhoub et al., 2006], whereas others showed no effect of calcitriol or paricalcitol on phosphate-induced SMC calcification [Wolisi and Moe, 2005; Wu-Wong et al., 2006], or effects by one but not another vitamin D sterol [Cardus et al., 2007].

Calcimimetics are allosteric small molecule activators of the calcium sensing receptor (CaSR) predominantly expressed on parathyroid glands. Calcimimetics, which decrease PTH and serum Ca and P levels in dialysis patients [Block et al., 2004; Lindberg et al., 2005], did not appear to induce VC in a rodent uremic model [Henley et al., 2005]. Whether this lack of calcification was the result of direct or indirect effects of calcimimetics on vascular cells could not be assessed in vivo. Some have described calcimimetic responses in SMCs in the absence of the parathyroid CaSR, whereas others have described the CaSR in SMCs and effects on mineralization [Farzaneh-Far et al., 2000; Smajilovic et al., 2006; Molostvov et al., 2007; Alam et al., 2009; Ivanovski et al., 2009]. In vitro, matrix gla protein (MGP) reporter activity increased in cultured rat SMCs in response to calcium and CaSR agonists [Farzaneh-Far et al., 2000] and this response was attributable to a CaSR-like receptor, rather than the CaSR. Others demonstrated calcium- and CaSR agonist-induced responses, CaSR mRNA and/or protein in rat and human SMCs and delay or inhibition of mineralization after calcimimetic R-568 exposure [Smajilovic et al., 2006; Molostvov et al., 2007; Ivanovski et al., 2009; Alam et al., 2009]. However, other reports showed no effect of calcimimetics on phosphate-induced calcification in bovine aorta SMCs [Shalhoub et al., 2006] and undetectable CaSR transcripts [Farzaneh-Far et al., 2000; Shalhoub et al., 2006]. Thus, reports on the presence of the CaSR and response to calcimimetics in SMC are conflicting, limiting the assessment of direct effects of calcimimetics on SMCs. Thus, we felt examining the effects of calcimimetics in the presence or absence of the CaSR warranted.

To elucidate possible interrelationships between mineral imbalance and secondary HPT therapeutics, we determined the gene expression profile in response to calcitriol, paricalcitol, and the calcimimetic R-568 in an in vitro model of human coronary artery SMC (CASMC) calcification. Vitamin D sterols exacerbated phosphate-induced calcification in human CASMC, while modulating multiple genes containing vitamin D receptor (VDR) response elements that were representative of multiple gene networks including mineralization, apoptotic, osteo/chondrocytic, and cardiovascular pathways. In contrast, the calcimimetic R-568 did not affect CASMC calcification or gene expression. Future studies will dissect which vitamin D-regulated genes are involved in protective and pro-calcification processes. These results may have implications for CKD-MBD patients on dialysis that are at increased VC risk and may be exposed to more than optimal phosphate and vitamin D sterol concentrations.

## MATERIALS AND METHODS

### Materials

Tissue culture plates were purchased from Falcon-Becton Dickinson (Franklin Lakes, NJ), phenol-red free Dulbecco's modified Eagle's medium (DMEM) from Cambrex Biosciences (Walkersville, MD); penicillin/streptomycin/glutamine, sodium pyruvate, and fungizone from GIBCO-Invitrogen (Grand Island, NY), fetal bovine serum (FBS) from Terra Cell (Etobicoke, Canada), calcitriol from Calbiochem-EMD Biosciences (La Jolla, CA), and paricalcitol from Abbott (North Chicago, IL). MILLIPLEX™ MAP kits to measure transforming growth factor beta (TGFβ2) and hepatocyte growth factor (HGF) were obtained from Millipore (Billerica, MA).

### Cells

Primary human CASMC from two donors (catalog number CC-2583: lot number 3F0246, donor 1; and lot number 00077, donor 2) were obtained from Clonetics (Walkersville, MD). Cell cultures were expanded in “expansion medium” (D-MEM/15% FBS/1 × Na pyruvate/penicillin/streptomycin; EM) as previously reported [Shioi et al., 1995]. CASMC from donor 1, but not donor 2, mineralized in EM in the presence of 10 mM beta-glycerolphosphate (BGP) within 2 weeks, and was used for subsequent studies. All experiments were performed on passage 7 cells, and results are from the average of 2–3 independent experiments. Cells for each dose–response and microarray experiment were plated at the same time from the same batch of cells, but in different size wells: for calcium accumulation dose–response (in 24 wells, n = 4); for microarrays (in 6 wells, n = 1). Thus, three independent experiments were performed sequentially, at different times, each with a dose–response and microarray component, so that gene expression could be correlated to calcification.

### Treatment Protocol for CASMC Calcification With Vitamin D Sterols and Calcimimetic R-568

Cells were seeded in 24-well plates (3,500 cells/cm^2^) in EM. Each experiment was performed in duplicate on separate plates (n = 2 wells/plate/concentration; thus n = 4 wells total/concentration [n = 3 independent experiments]). Culture conditions were chosen based on those for optimal conversion of vascular SMC [Mori et al., 1999] or mesenchymal stem cell [Cheng et al., 1994] into osteoblastic cells. Ascorbic acid (AA) is a cofactor for collagenous extracellular matrix formation (not present in DMEM), while BGP provides a phosphate source after alkaline phosphatase (ALP) enzymatic phosphate release. The glucocorticoid dexamethasone (Dex) accelerates phosphate-induced CASMC calcification [Mori et al., 1999], therefore a concentration of 10^−9^ M Dex was included in differentiation medium (DM) comprised of expansion medium, AA (50 mg/ml), Dex (10^−9^ M), and BGP (10 mM) [Shioi et al., 1995; Mori et al., 1999]. Expansion medium with AA and Dex is referred to henceforth as “basic medium, BM.” For vitamin D sterol and calcimimetic exposure, DM was supplemented with vehicle (vehicle + DM), calcitriol (10^−11^–10^−7^ M) (calcitriol + DM), paricalcitol (10^−11^–10^−7^ M) (paricalcitol + DM), or R-568 (10^−11^–10^−7^ M) (R-568 + DM). Although three different vehicles were used (100% ethanol [calcitriol], 30% polyethylene glycol/20% ethanol in water [paricalcitol], and distilled water [R-568]), no vehicle-induced differences in gene expression were observed. Cell medium was changed every 2–3 days for a total of three feedings over 7 days.

### Treatment Protocol for CASMC Calcification With Calcium and Phosphate

Cells were cultured in 24-well plates (3,500 cells/cm^2^, n = 4) in EM. After 5 days CASMC were exposed to BM or BM adjusted with increasing concentrations of total calcium (in the form of CaCl_2_) and/or phosphate (in the form of BGP). The final concentration in BM of total calcium was 1.8, 2.8, and 3.6 mM and phosphate was 0.9, 2, 4, 5 mM. Cell medium was changed every 2–3 days for a total of three feedings over 7 days. In DMEM calcium and phosphate concentrations are 1.8 and 0.9 mM, respectively.

### Mineralization (von Kossa) Staining

Mineral deposits were detected with von Kossa staining by incubating cultures with 3% silver nitrate (Sigma) for 30 min under ultraviolet light as previously described [Shalhoub et al., 2006].

### Quantitative Assay for Calcium Accumulation

Calcium was quantified as previously described [Mori et al., 1999]. Briefly, cell layers were washed with Tris buffer (10 mM, pH 7.0) prior to Ca solubilization with 0.6 N HCl. Ca concentration was determined by cresolphthalein procedure (Stanbio Laboratory, Boerne, TX), normalized to protein content from the same well. Protein was solubilized with 0.1 N NaOH/0.1% SDS and quantified by the Coomassie Blue method (Pierce, Rockford, IL). Calcium is therefore expressed as micrograms Ca/mg protein.

### RNase Protection Assays

Total RNA was extracted from CASMC using STAT60 reagent (Tel-test, Friendswood, TX) and quantified as previously described [Shalhoub et al., 2006]. Radiolabeled antisense RNA probes were transcribed from linearized plasmid template using RNA polymerase and a ^32^P-uridine triphosphate (UTP). RNase protection assays were performed using the RPA II kit (Ambion, Austin, TX) and 5 µg (ALP, Runx2) or 10 µg (CaSR, VDR) of total cellular RNA. After overnight exposure, phosphor screens were scanned and densities of the protected bands were calculated with ImageQuant software (GE Healthcare Bio-Sciences Corp., Piscataway, NJ). The GenBank # and size of protected fragment for the examined genes were: CaSR (U20759 nts 1786–2234), VDR (J03258.1 nts 192–376), ALP (X14174 nts 450–685), Runx2 (NM_004348 nts 853–1045).

### Microarray Assays

For microarray studies, CASMCs were cultured in parallel with dose–response experiments using the same batch of cells. Passage 7 cells grown in 6-well dishes at 3,500 cells/cm^2^ were exposed to vehicle + DM alone, calcitriol (10^−8^ or 10^−11^ M) + DM, paricalcitol (10^−8^ or 10^−11^ M) + DM, or R-568 (10^−7^ or 10^−11^ M) + DM for 4 h or 1, 3, or 7 days (n = 1 well per treatment, three independent experiments). Results were the average of three independent experiments. Total RNA was isolated using Qiagen RNeasy Mini Kit (Valencia, CA) and processed following the protocols described in section 2 (*Eukaryotic Sample and Array Processing*; 701024 rev 1) of the Affymetrix Technical manual. Briefly, 5 µg total RNA was used to synthesize cDNA (10 pmol of T7-(dT)_24_ primer, and Superscript II (Invitrogen, Carlsbad, CA). Purified double-stranded cDNA (MinElute Reaction Cleanup Kit (Qiagen, Valencia, CA)) was used to generate biotinylated cRNA (Bioarray High Yield RNA Transcript labeling Kit (Enzo Diagnostics, Farmingdale, NY); purified by Qiagen RNeasy Mini kit) for hybridization to the Affymetrix Human Genome U133 Plus 2.0 array. Arrays were washed on a GeneChip Fluidic Station 450 (EukGE_WS2v4_450 protocol) and scanned using the Affymetrix GeneChip Scanner 3000 (Affymetrix, Santa Clara, CA). Data can be accessed at http://www.ncbi.nlm.nih.gov/geo/query/acc.cgi?acc=GSE11917.

### Branched DNA (bDNA) Analysis

Changes in expression of select genes were confirmed by bDNA analyses using the QuantiGene Screen Kit (Genospectra, Fremont, CA) according to the manufacturer's instructions. Expression values normalized to cyclophilin levels were log_2_ transformed.

### Protein Analysis of Culture Medium

After the last feeding, the 48-h conditioned culture medium (with different concentrations of vitamin D sterols or R-568) was transferred at harvest on day 7, and frozen at −80°C for later protein determination. Transfer involved pooling each two of n = 4 wells (1 ml DM/well) from duplicate plates. This resulted in two, 2 ml samples per treatment. Each point on dose–response curves represents results from one, 2 ml pooled sample, from one out of two experiments, each performed in duplicate. The medium was thawed on ice and TGFB2 and HGF levels were measured using the sensitive MILLIPLEX™ MAP assay, which is based on LuminexR xMAP technology (Millipore). Each kit contained two quality control samples, a low range (146–303 ng/ml) and high range (558–1,158 ng/ml) for TGFB2; and a low range (546–1,595 pg/ml) and high range (2,896–7,888 pg/ml) for HGF. The quality control sample values for TGFB2 and HGF were verified within the expected ranges, validating assay reagents and results obtained with conditioned medium.

### Statistics

Values are presented as mean ± SE. Significance (*P* < 0.05) was tested using an ANOVA with a Fischer's post hoc test (calcification) or an ANOVA with a Bonferonni correction for multiple testing (as indicated) (gene expression).

For the microarray analysis, statistics were performed as previously described [Shalhoub et al., 2006], with the addition of R-568 + DM group. “Perfect match” oligonucleotide reporters used to determine intensity measurements were log-transformed, scaled using a nonlinear normalization, summarized into a single intensity using the arithmetic mean and referred to as “gene intensities.” For error-weighted ANOVA, the reciprocal of the squared error was used as a weight. Intensities reported as zero or otherwise flagged by the image analysis software were treated as missing. Hierarchical clustering was performed using the correlation matrix as a measure of similarity. Linkage maps were built using the single-linkage method.

ANOVA was performed on a subset of genes to test the null hypothesis that gene intensity was not altered due to treatment group. In tests involving multiple runs, experimental run number was treated as a random covariate and an error weighted ANOVA was performed using SAS/JMP (Cary, NC). Samples were divided into the following groups: EM + vehicle, BM + vehicle, DM + vehicle, calcitriol (10^−8^ or 10^−11^ M) + DM, paricalcitol (10^−8^ or 10^−11^ M) + DM, or calcimimetic R-568 (10^−7^ or 10^−11^ M) + DM.

Significantly changed genes at day 7, as well as candidate genes not changed at day 7, were examined for significant changes at earlier times (4 h, and 1 and 3 days). It is possible that additional genes were significantly changed at the earlier times.

### Vitamin D Response Element (VDRE) Promoter Analysis

Upstream promoter sequences of the regulated genes identified in this study were extracted from UCSC genome server (http://genome.ucsc.edu/) [Karolchik et al., 2004], which contains sequences −2,500 bp upstream of the transcription starting site. The position weight matrix (PWM) of VDRE was obtained from JASPAR [Sandelin et al., 2004], an open-access database of annotated eukaryotic transcription factor binding profiles. The VDRE binding sites in the promoter regions were predicted with the PATSER computer program in bioperl [Stormo et al., 1982; Letondal, 2001; Stajich et al., 2002], a programming interface for bioinformatics application programmers, and significant putative VDRE sites were selected with the PATSER score cut-off >75. This score is assumed to be exponentially related to the probability of binding. PATSER also calculates a *P*-value cut-off for each weight matrix using the information content [Staden, 1989].

## RESULTS

### Calcification of Human Donor CASMCs

**Fig. 1:**
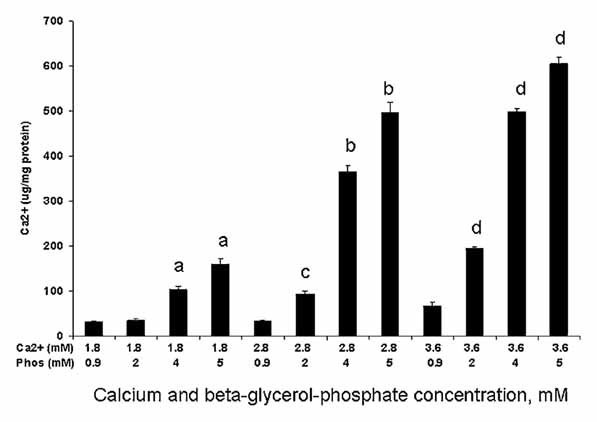
Elevated extracellular calcium and/or phosphate enhanced CASMC mineralization. CASMC were culture expanded for 5 days before exposure to different phosphorus (beta-glycerol-phosphate) and total calcium concentrations. Synergistic increase in calcification with combination of calcium and phosphorus. ^a^*P* < 0.0001 versus 1.8/1.9 mM Ca/P; ^b^*P* < 0.0001 and ^c^*P* < 0.001 versus 2.8/0.9 mM Ca/P; ^d^*P* < 0.0001 versus 3.6/0.9 mM Ca/P.

We sought to determine the gene expression changes associated with vitamin D sterol enhancement of VC induced by uremic-like conditions found in secondary HPT using an in vitro model of human CASMC calcification. To properly model the calcification of human vascular SMC induced by the mineral imbalance found in CKD-MBD patients [Reynolds et al., 2004; Yang et al., 2004], calcification in response to increased calcium and phosphorus concentrations was monitored. A 7-day exposure to BM with elevated concentrations of total calcium and phosphorus increased the calcification of CASMCs (Fig. 1). This Ca- and P-induced calcification was dose-dependent and synergistic (Fig. 1).

**Fig. 2:**
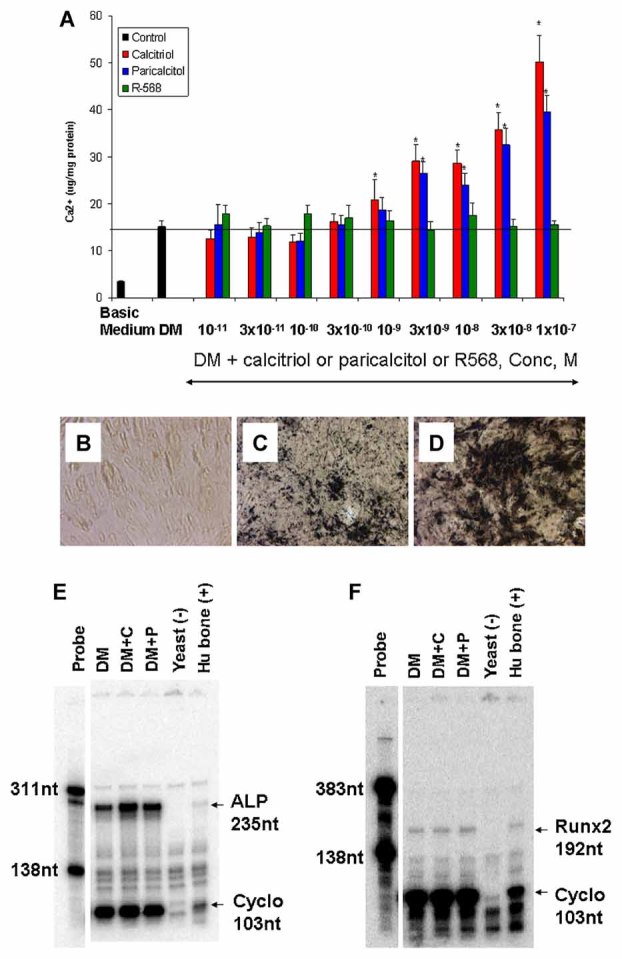
Calcitriol + DM and paricalcitol + DM, but not R-568 + DM, dose dependently increased calcification of human CASMC. **A**: CASMC were culture expanded for 5 days and exposed to DM supplemented with 10^−11^–10^−7^ M calcitriol, paricalcitol or R-568 for an additional 7 days. Calcium was determined and normalized to protein as in the Materials and Methods Section. *P < 0.05 versus DM. Results represent the average of two independent experiments. **B**–**D**: Representative von Kossa stained CASMC cultures showing enhanced calcification by vitamin D sterol + DM compared with DM. CASMC were culture expanded for 5 days and then exposed for 7 days to basic medium + vehicle (B), vehicle + DM (C), or calcitriol (10^−7^ M) + DM (D) (20× magnification). **E**: Ribonuclease protection assay demonstrated an increase in alkaline phosphatase mRNA in 7-day cultures exposed to calcitriol + DM (10^−8^ M) and DM+ paricalcitol (10^−8^ M). The protected fragment was 235 nt. E: Ribonuclease protection assay demonstrated Runx2 mRNA expression in 7-day CASMC cultures. The protected fragment was 192 nt.

In contrast to the lack of calcification in human CASMC cultured in BM containing AA (50 mg/ml), Dex (10^−9^ M), Ca (1.8 mM), and P (0.9 mM) (BM) for 7 days (Fig. 2A,B), there was pronounced black von Kossa stained mineral deposits in the extracellular matrix of CASMC exposed to 10 mM BGP (DM) (Fig. 2C). Calcification in the presence of DM was quantified in Figure 2A.

### Calcification of CASMCs in the Presence of Phosphate and Vitamin D

The addition of vitamin D sterols (calcitriol or paricalcitol) enhanced the calcification of CASMCs induced by 7 days of DM [AA (50 mg/ml), Dex (10^−9^ M), Ca (1.8 mM), and BGP (10 mM)] (Fig. 2A). The von Kossa mineral staining induced by DM was enhanced by exposure to calcitriol (10^−7^ M) + DM (Fig. 2D) or paricalcitol (10^−7^ M) + DM (not shown) for 7 days. This enhanced calcium accumulation was dose-dependent over the range of 10^−11^–10^−7^ M vitamin D sterol (calcitriol EC_50_ 9.9 ± 0.3 nM, paricalcitol EC_50_ 8.7 ± 0.3 nM) (Fig. 2A). There was no effect on calcium accumulation in response to the calcimimetic R-568 (10^−11^–10^−7^ M; 7 days) + DM (Fig. 2A). To further confirm the utility of the model cell system used, we sought to establish the expression and vitamin D sterol-induced regulation of two well-characterized mineralization genes in the CASMCs. CASMCS cultures expressed both ALP and runt related transcription factor 2 (Runx2) mRNA (Fig. 2). Whereas ALP mRNA expression was increased by calcitriol + DM (Figure 2E) and paricalcitol + DM (Fig. 2E), Runx2 mRNA expression was unchanged (Fig. 2F).

### Microarray Analyses

To test the hypothesis that the enhanced CASMC calcification was associated with altered expression of genes we sought to identify genes differentially regulated by vitamin D sterols or R-568. Microarray analyses were performed with total RNA isolated from cultures exposed to vehicle + DM or vitamin D sterol (10^−8^ or 10^−11^ M) + DM or R-568 (10^−7^ or 10^−11^ M) + DM for 4 h and 1, 3, and 7 days. The concentrations of vitamin D sterols were based on the physiological concentration of 1,25 dihydroxyvitamin D_3_ (10^−10^–10^−11^ M) and the supraphysiological concentration (10^−8^ M) commonly used in vitro to examine gene expression that effectively enhanced DM-induced calcification. In dialysis patients, calcitriol (5–35 µg/dialysis session) is administered to achieve normal blood concentrations of 40–80 pg/ml (=1–2 × 10^−10^ M); and paricalcitol is administered at fivefold higher doses. The R-568 concentrations were based on those found in patient sera (C_max_ of 7.7 ng/ml, ∼2.3 × 10^−8^ M R-568) [Goodman et al., 2000a; Shalhoub et al., 2006]. The genes identified with significantly (*P* < 0.05) altered expression in medium containing AA + Dex (BM) compared with EM, and AA + Dex + BGP (DM) compared with BM after 7 days of treatment, are reported in Supplemental Tables I–III. The effect of Dex in the absence of AA was not assessed.

This analysis identified 53 genes that were significantly (*P* < 0.05 as compared to DM alone) upregulated by a 7-day exposure to calcitriol (10^−8^ M) + DM exposure with fold changes ranging from 1.06 to 10.1 (Supplemental Table I). There were 35 genes significantly downregulated by −1.07- to −1.84-fold following 7 days of calcitriol + DM (Supplemental Table II). For most genes, induction and repression patterns with calcitriol + DM and paricalcitol + DM were virtually identical for significantly regulated genes (Tables I–IV; Supplemental data, Tables I–III).

**TABLE I:** Calcitriol (10^−8^ M) + DM and Paricalcitol (10^−8^ M) + DM Significantly Modulated Genes Involved in Human Cardiovascular and Skeletal Function,* Compared With DM

Gene	Calcitriol fold change	Paricalcitol fold change	Skeletal disorder	Cardiovascular disorder
BMP4 (7d)	**+1.3**	*+1.1*	Polymorphisms associated with hip BMD	
BMP6 (7d)	**+1.3**	**+1.1**	Rheumatoid arthritis	Found in intimal plaque SMCs
TNFRSF11B (OPG) (7d)	**+1.3**	**+1.3**	Idiopathic hyperphosphatasia (Juvenile Paget's disease)	Elevated in serum of CKD patients and in calcified vessels
CILP (7d)	**+1.5**	**+1.2**	SNIP associated with lumbar disc; crystal deposition in joint disease	
IL-6 (3d)	**+1.3**	*+1.1*		Cardiovascular disease
KCNK3 (7d)	**+1.5**	*+1.2*		Arrhythmia
DSP (7d)	**+1.3**	**+1.2**		Arrhythmogenic ventricular cardiomyopathy
THBD (1d)	**+1.4**	**+1.3**		Coronary heart disease
ENPP1 (7d)	**−1.3**	**−1.2**	Periarticular calcification	Spontaneous infantile arterial calcification
TNC (7d)	**−1.3**	*−1.1*	Polymorphism in Achilles tendon injury	Hypertension. Left ventricular remodeling.
GCLC (3d)	**1.4**	**1.2**		Endothelial vasomotor dysfunction; myocardial infarction
ASPN (7d)	*−1.4*	*−1.3*	Susceptibility to arthritis	
HGF (7d)	**−1.8**	**−1.4**		Hypertension/atherosclerosis/cardiovascular disease
S100A4 (3d)	**−1.5**	**−1.4**	Arthritis	Plexogenic arteriopathy in pulmonary hypertension
CLEC3B (7d)	**−1.7**	**−1.4**	Rheumatoid arthritis	

Affymetrix probe set numbers can be found in Supplemental data, Tables I and II.

BMP, bone morphogenetic protein; OPG, osteoprotegerin; CILP, cartilage intermediate layer protein nucleotide pyrophosphohydrolase; IL-6, interleukin-6; KCNK3, potassium channel, subfamily K, member 3; DSP, desmoplakin; THBD, thrombomodulin; ENPP1, ectonucleotide pyrophosphatase/phosphodiesterase 1; TNC, tenascin C; ASPN, asporin; TGFBR2, transforming growth factor beta receptor 2; HGF, hepatocyte growth factor; S100A4, S100 calcium binding protein A4; CLEC3B, C-type lectin domain family, member B; GCLC, glutamate-cysteine ligase, catalytic subunit.

* Values are for the measurement of maximal change (4 h, 1, 3, or 7 days) for calcitriol (10^−8^ M) + DM. Bold: *P* < 0.001; italics: *P* < 0.05.

**TABLE II:** Calcitriol and Paricalcitol Significantly Modulated Genes Involved in Mouse Cardiovascular and Skeletal Function* Compared With DM

Gene	Calcitriol fold change	Paricalcitol fold change	Skeletal disorder	Cardiovascular disorder
CYP24A1 (3d)	**+11.1**	**+5.5**	Abnormal intramembranous bone formation	
Dkk1 (7d)	*+1.4*	*+1.3*	Fused vertebrae, postaxial polysyndactyly	
HHIP (7d)	**+1.3**	**+1.2**	Controls hedgehog proteins in cartilage; Over-expression leads to short skeleton like Ihh knockout	
TGFB2 (3d)	**+1.8**	**+1.5**	Craniofacial chondrogenesis/defects in bones	Cardiovascular defects
DSP (7d)	**+1.3**	**+1.2**		Lethal heart defects, ventricular dysfunction, myocyte apoptosis, larger ventricles
Wnt5A (3d)	**−1.3**	*−1.3*	Chondrocyte differentiation defect; lack of limb outgrowth	Congenital heart disease; truncus arteriosus
CLEC3B (7d)	**−1.7**	**−1.4**	Spinal deformity	
THBS1 (7d)	*−1.6*	**−1.1**		Thrombus in injured vessel

Affymetrix probe set numbers can be found in Supplemental data, Tables I and II.

CYP24A1, cytochrome P450, family 24, subfamily A; Dkk1, Dickkopf-related protein 1; HHIP, hedgehog interacting protein; TGFB2, transforming growth factor beta 2; DSP, desmoplakin; Wnt5A, wingless-type MMTV site family member 5A; CLEC3B, C-type lectin domain family, member B; THBS1, thombospondin.

* Mouse phenotypes are described for genes, some of which lack direct human disease data. Values are for the measurement of maximal change (4 h, 1, 3, or 7 days) for calcitriol (10^−8^ M) + DM as compared to DM. Bold: *P* < 0.001: italics: *P* < 0.05.

**TABLE III:** Other Calcitriol (10^−8^ M) + DM and Paricalcitol (10^−8^ M) + DM Significantly Modulated Genes* Compared With DM

Gene	Calcitriol fold change	Paricalcitol fold change	Gene function family
NLRP1 (3d)	**+1.4**	**+1.2**	Apoptosis
GIP3 (7d)	**−1.8**	**−1.7**	Cytokine
COL22A1 (3d)	**+1.4**	**+1.2**	Extracellular matrix
MAPK13 (3d)	**+1.3**	**+1.1**	Cell cycle/signal transduction
SLC22A3 (3d)	**+2.1**	**+1.8**	Cationic channel/transporter
GCLC (3d)	**+1.4**	**+1.2**	Anti-oxidant

Affymetrix probe set numbers can be found in Supplemental data, Tables I and II.

NLRP1, NACHT-leucine-rich-repeat and PYD-(pyrin-domain)-containing-1; GIP3, interferon, alpha-inducible protein; COL22A1, collagen type 22, alpha 1; MAPK13, mitogen-activated-protein-kinase 13; SLC22A3, solute-carrier-family-22; GCLC, glutamate-cysteine ligase, catalytic subunit.

* Values are at the measurement of maximal change (4 h, 1, 3, or 7 days) for calcitriol (10^−8^ M) + DM. Bold: *P* < 0.001.

**TABLE IV:** Branched DNA (bDNA) Analysis of Human CASMC Gene Expression After 7 Days of DM, Calcitriol + DM or Paricalcitol + DM Exposure

	Fold change	
	
Gene	Cal + DM vs. DM	Par + DM vs. DM
Alkaline phosphatase (ALP)	**1.47**	1.31
Matrix gla protein (MGP)	1.08	−1.10
Fibrillin 1 (FBN1)	1.04	1.08
Osteoprotegerin (TNFRSF11B)	**2.13**	**1.82**
Secreted frizzled related protein-3 (FRZB)	−1.25	−1.08
Ectonucleotide pyrophosphatase/phosphodiesterase 1 (ENPP1)	**−1.52**	−1.40
Runt related transcription factor 2 (RUNX2)	1.04	−1.09

Cal, calcitriol; Par, paricalcitol; DM, differentiation medium.

The data were normalized to cyclophilin levels and then were transformed using log_2_.

The values displayed here are fold change (or negative reciprocal if fc < 1) of the treatment 1–treatment 2, where treatments 1 and 2 are given as column names.

Bold values indicate fold changes are not equal to 1 (*P* < 0.05).

When assessing the significantly altered genes using a 1.3-fold change threshold for any one time point and any one treatment, 31 genes were upregulated with fold changes ranging from 1.3 to 10.1 and 14 genes were downregulated with fold changes of −1.3 to −1.84 (Supplemental Tables I and II). For Tables I and II in Results Section, treatment-induced gene expression alterations were identified as those genes that were significantly (*P* < 0.05) and substantially (fold change >1.3) altered at any time point following any treatment. Thus, if a gene was found significantly elevated at day 7 for calcitriol + DM the corresponding value for the same time point for paricalcitol + DM (even though not significant) was included in Tables I and II for comparison. Supporting the validity for a 1.3-fold cut-off, the majority of genes identified with altered expression by 7 days of exposure had expression changes in the same direction at one or more of the earlier time points (Supplemental Tables I and II). In contrast to the significant effects observed with the supraphysiological concentrations of vitamin D sterols, the 10^−11^ M concentration had no effect on calcification or gene expression in vitro (data not shown). In general, when administered at the same concentration (10^−8^ M), calcitriol exerted a more pronounced effect on CASMC gene regulation than paricalcitol (e.g., CYP24A1 mRNA increased 11.1- and 10.1-fold with calcitriol and 5.5- and 3.9-fold with paricalcitol at 3 and 7 days, respectively). In pre-clinical studies, calcitriol was about threefold more potent compared with paricalcitol to decrease PTH secretion from parathyroid glands [Slatopolsky, 1998]; and, clinically, a three to fivefold higher dose of paricalcitol is administered to achieve the same lowering effect on PTH secretion. In contrast to the altered gene expression profiles in response to vitamin D sterols (10^−8^ M), no significant changes were observed with R-568 (10^−7^ M) + DM. ALP expression was significantly elevated on microarrays for both vitamin D sterols + DM (*P* < 0.05) (Supplemental Table I).

Genes identified as being modulated by Vitamin D sterol + DM are involved in multiple cellular processes, including vitamin D metabolism, apoptosis, cell cycle, mineralization and cardiovascular pathways (Supplemental Tables I and II). Focusing on genes with a high probable involvement in VC, we identified the time point of maximal fold change (calcitriol + DM or paricalcitol + DM as compared to DM alone) for the significantly regulated genes with known skeletal or cardiovascular function in human (Table I) and mouse (Table II). As a positive control for vitamin D sterol effect we used the vitamin D metabolizing enzyme, cytochrome P450, family 24, subfamily A (CYP24A1), which is upregulated in most vitamin D target tissues in response to vitamin D [Kato et al., 2007]. CYP24A1 mRNA showed an early response (by 4 h), and was the most dramatically changed of all the transcripts (Table I and Supplemental Table I). Other genes significantly regulated by 4 h were the potassium channel subfamily K, member 3 (KCNK3), the antioxidant, glutamate-cysteine ligase, catalytic subunit (GCLC) and thrombomodulin (THBD).

### Gene Expression Confirmation by bDNA Analysis and Protein Expression

**Fig. 3:**
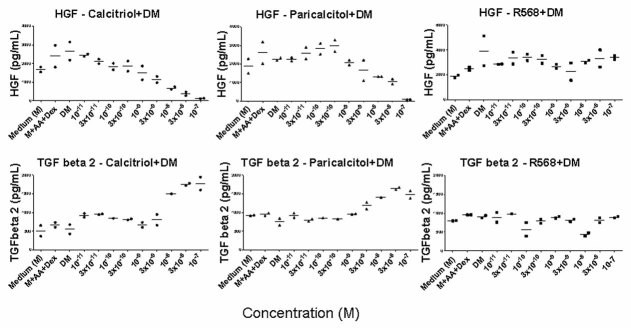
Protein analysis in culture medium of human CASMCs after 7 days of DM, calcitriol + DM or paricalcitol + DM exposure confirms microarray profile. Upper three panels: HGF after calcitriol, paricalcitol and R-568 exposure, respectively. Lower three panels: TGFβ2 after calcitriol, paricalcitol and R-568 exposure, respectively. Each point on the dose–response curves represents results from one out of two experiments. Each experiment was performed on duplicate plates (n = 2 wells/plate/concentration); thus n = 4 wells total/concentration. Each concentration was assayed in duplicate wells (after n = 4 wells were pooled into n = 2 wells), hence the two points per concentration on graphs. M + AA + DEX + BG*P* = DM.

To validate the gene expression changes detected by in the above analyses, bDNA analysis was performed for a subset of identified genes to include genes believed to be involved in calcification that were upregulated, downregulated, and unchanged by exposure to vitamin D sterols in DM. The upregulated gene set included ALPL and tumor necrosis factor (receptor) superfamily member 11B (TNFRSF11B, also known as osteoprotegerin, OPG). The downregulated gene set included secreted frizzled related protein 3 (FRZB), MGP and ectonucleotide pyrophosphatase/phosphodiesterase 1 (ENPP1). The unchanged gene set included fibrillin 1 (FBN1) and Runx2. The expression patterns determined by bDNA analysis were similar to those identified by the microarray analysis (Table IV, Supplemental Tables I–III). Of note Runx2 expression was not altered by AA + DEX or DM, as seen with the microarray analysis. In addition, protein expression at day 7 was determined for TGFβ2 (upregulated) and HGF (downregulated) (Fig. 3) after exposure to calcitriol + DM, paricalcitol + DM, and R-568 + DM. The protein expression patterns for HGF and TGFβ2 were consistent with those identified by the microarray analysis (Tables I and II).

### Vitamin D Response Element Analysis

**Fig. 4:**
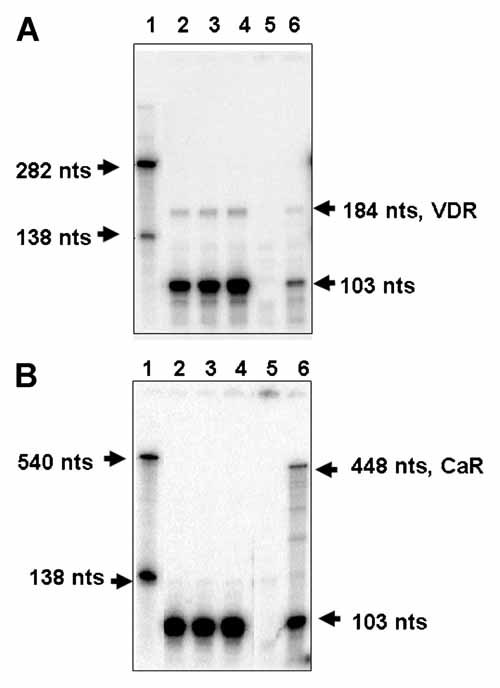
Human CASMCs express vitamin D receptor (VDR), but not calcium sensing receptor (CaSR), mRNA by RNase protection assays. **A**: The 282 nt VDR probe protected a 184 nucleotide (nt) fragment. **B**: The 540 nt CaR probe protected a 448 nt fragment. The 138 nt cyclophilin (cyclo) probe protected a 103 nt fragment. Lane 1: Probe; lanes 2 and 3: CASMC lot number 3F0246; lane 4: CASMC lot number 00077; lane 5: yeast (negative control); lane 6: kidney (positive control).

To identify possible mechanism of transcriptional regulation of genes associated with CASMC calcification we sought to locate putative VDREs in the promoters of regulated genes and to determine the VDR expression of CASMC. Promoter analyses demonstrated that the promoters of all of the regulated genes identified contained at least one putative VDRE (Supplemental Tables I and II). RNase protection assays with total RNA isolated from culture expanded primary CASMC confirmed the presence of VDR mRNA in CASMCs (Fig. 4A). In contrast, we were unable to identify CaSR transcripts in CASMC (Fig. 4B). Both VDR and CaSR transcripts were present in RNA isolated from kidney (positive control), but not yeast (negative control) (Fig. 4). The absence of CaSR mRNA was consistent with lack of CASMC response to calcimimetic R-568.

## DISCUSSION

In the present study, we tested the hypothesis that enhanced CASMC calcification is associated with altered expression of genes under transcriptional regulation by vitamin D sterols. Consistent with this hypothesis, mineral deposition by CASMC in the presence of phosphate-containing DM was enhanced in a dose-dependent manner by calcitriol and paricalcitol over 7 days. This vitamin D sterol-enhanced calcification was correlated with CASMC gene expression changes which began by 4 h after initial exposure, confirming that vitamin D sterols have direct effects on SMC. Although most fold changes in gene expression obtained after exposure to vitamin D sterols + DM, compared with DM, were modest (*P* < 0.001; Tables I–III), they were significant at more than one time point (Supplemental Tables I and II). The modest fold changes were not unexpected given the vitamin D concentration (10^−8^ M) administered in vitro in this study. The gene changes may have been greater with higher vitamin D sterol doses than used here [Wu-Wong et al., 2007a] or in the absence of dexamethasone, which is known to antagonize vitamin D effects in some systems. Although the fold change was generally not greater than 2, it is likely that the totality of gene changes induced by vitamin D sterol + DM treatment, tipped the balance towards the calcification phenotype, with some protective against calcification, and others supporting calcification.

The present study is consistent with some, but not all, past reports of increased phosphate-enhanced calcification of bovine ASMC (BASMC) by vitamin D sterols. In bovine SMC, calcitriol [Shalhoub et al., 2006] and paricalcitol [Shalhoub et al., 2006] increased phosphate-induced calcification and chondro/osteoblastic gene expression. Other studies reported either no effect of calcitriol or paricalcitol on phosphate induced bovine [Wolisi and Moe, 2005] and human SMC calcification [Wu-Wong et al., 2006], or calcitriol-, but not paricalcitol-, induced calcification in rat SMC [Cardus et al., 2007]. Future studies are needed to clarify if differences in experimental design or model systems underlie these conflicting reports.

The lack of effect by R-568 observed in the current study is consistent with the lack of CaSR expression in these CASMCs and with previous reports in bovine ASMC. Although CaSR mRNA was not detected in this study, CaSR mRNA and/or protein and calcimimetic responses were demonstrated in rat and human SMC by others [Farzaneh-Far et al., 2000; Smajilovic et al., 2006; Molostvov et al., 2007; Ivanovski et al., 2009; Alam et al., 2009]. Farzaneh-Far et al. showed that calcium and CaSR agonists induced MGP/promoter reporter expression in rat aorta SMCs. However, CaSR transcripts were not detected and this response was attributed to a separate CaSR-like receptor. Smajilovic et al. demonstrated CaSR mRNA (RT-PCR) and protein (immunocytochemical) in rat aortic SMC, and showed that calcium and neomycin induced proliferation, which was not inhibited by dominant-negative CaSR silencing. This suggested that other receptors/pathways for calcium and neomycin were operative to induce proliferation. Molostvov et al. demonstrated CaSR mRNA (RT-PCR) in human aortic SMCs; and, that responses to CaSR agonists were inhibited by knock-down CaSR-siRNA methods. In addition, Molostov et al. showed that CaSR expression declined with severity of kidney disease in patient vessels. Alam et al. demonstrated CaSR protein (immunohistochemistry) in VSMCs on human arterial sections and in human and bovine SMCs (immunofluorescence or Western blot) in vitro. Alam et al. also showed that the calcimimetic R-568 caused a delay in mineralization of human SMCs cultures, and upregulated CaSR. These experiments supported an anti-mineralization protective role for SMC CaSR. Ivanovski et al. demonstrated that the calcimimetic R-568 inhibited phosphate-induced human aortic SMC mineralization after 10 days in culture medium that contained 1% serum; and, silencing with CaSR-siRNA abrogated this calcimimetic effect. In the present study, we cannot explain why the human CASMCs and, previously, bovine aorta SMCs did not respond to calcimimetic exposure, other than absence of the CaSR. It is likely that different species, donors, culture systems, and assay methods account for these divergent results for the effects of calcimimetics on SMCs.

It is widely believed that VC is a highly regulated, cell-mediated, multi-factorial process associated with the expression of chondro/osseous genes and their protein products [Jono et al., 2000; Tyson et al., 2003]. Two commonly studied osteo/chondrogenic genes, Runx2 and osteocalcin (OC), have been shown to be upregulated in human fetal SMC upon exposure to elevated phosphorus concentrations [Jono et al., 2000]. In contrast, normal adult human SMCs often express osteo/chondrogenic genes, including osteocalcin and Runx2, spontaneously in vitro [Shanahan and Weissberg, 1999; Tyson et al., 2003]. Thus, Runx2 may be regulated differently in fetal and adult SMC [Jono et al., 2000]. In this study, we demonstrate that Runx2 and osteocalcin mRNA are expressed in our hCASMCs and their mRNA expression levels were not changed by vitamin D sterol + DM. Further, the expression of Runx2 protein was not changed by DM or vitamin D sterols (data not shown), confirming the findings in a previous report [Wu-Wong et al., 2007a]. Although the expression of these two osteo/chondrogenic genes was not changed, many other genes related to mineralization were altered in vitamin D sterol-treated CASMC resulting in a gene expression pattern indicative of a shift to a mineralizing osteoblast-like cell phenotype [Favus, 2006]. Such a shift is represented by concurrent increases in pro-mineralization gene expression (e.g., ALPL, TGFB2, BMP4 and 6) and decreases in anti-mineralization gene expression (ENPP1) along with changes in IBSP, OPG and the chondrocyte genes, ANXA3, CILP, TNC, ITGA8, and cytokine IL-6. Changes in the expression of pro- and anti-apoptotic genes are consistent with the proposed role of apoptosis as a calcification initiator in human CASMC [Reynolds et al., 2004]. In addition to these genes implicitly involved in mineralization, we also observed altered expression of genes normally expressed in heart or skeleton that are associated with cardiovascular or joint diseases including, DSP, THBS1, THBD and HGF, HGF binding protein (CLEC3B), ASPN, S100A4, SLC22A3, KCNK3, HHIP, Dkk1.

This study is limited by the model system used. This study was designed to determine the gene expression changes associated with vitamin D sterol enhancement of VC induced by uremic-like conditions found in secondary HPT. This precluded the use of cells from the second donor that lacked a calcification response to high P, even though they may have calcified in the presence of high P and vitamin D sterols. Therefore, these data can be considered representative of only those patients whose arteries might calcify in the presence of uremic concentrations of phosphate and not of all sHPT patients. Further the cell passage number and differentiation media used in the studies limit the ability to directly compare these results with other reports that used different cell growth conditions. Although the passage 7 cells, used in accordance with earlier studies of human SMCs [Steitz et al., 2001], responded to phosphate and vitamin D sterols, earlier passages were not assessed for calcification potential or gene expression profiles. Therefore we cannot rule out de-differentiation of the cells during expansion. The current experiments used medium containing AA and Dex (10^−9^ M), making comparisons with other studies, performed with EM + BGP alone, difficult due to possible confounding by the effects of these agents on VSMCs. Dexamethasone was used to shorten the mineralization time-line by several days due to its reported enhancement of SMC and human osteoblast mineralization [Cheng et al., 1994; Mori et al., 1999]. However, the inclusion of AA + Dex could, in part, explain differences between CASMC and bovine aorta SMC results [Shalhoub et al., 2006] due to known effects of Dex on differentiation, migration, proliferation, and gene expression profiles in VSMCs [Mori et al., 1999]. Although these differences may also be explained, in part, by species differences or use of different passage numbers, some bovine aorta SMC genes modulated after 7 days treatment [Shalhoub et al., 2006] (MGP, FRZB, IL-6, COLXIA1) were changed in the same direction in this study, albeit some at earlier times (Supplemental Tables I and II). In vivo validation of these in vitro findings is required for regulated genes not previously reported at sites of vitamin D-induced calcification.

Results presented here provide novel insights regarding mechanisms that may be involved in regulating vitamin D sterol-mediated calcification of human CASMC. In CASMCs expressing VDR, vitamin D sterol-enhanced calcification is associated with altered expression of genes whose promoters contain VDRE. Thus, vitamin D sterols likely influence transcription in these non-classical vitamin D target cells via genomic pathways with enhancement or suppression of transcription rates reflecting recruitment of different co-regulators to the VDR transcriptional complex [Dusso et al., 2005]. However, non-genomic pathways cannot be ruled out and future studies will be required to determine if VDR interacts with the putative VDREs.

The effect of vitamin D sterols on gene expression in the absence of added phosphate was not addressed in this study due to the lack of calcification using these culture conditions in our laboratory (previous unpublished observations). Others have demonstrated that in resting human CASMCs (no added phosphate), vitamin D modulates genes including some identified in this study (ENPP1, THBD, and THBS1) [Wu-Wong et al., 2007b], but association with calcification was not examined. Future studies will be focused on determining whether vitamin D sterol regulation of cardiovascular and osteoblastic genes is dependent upon existing mineral disturbances or prior phenotypic switch.

In conclusion, we identified altered gene expression patterns accompanying vitamin D sterol-induced exacerbation of human CASMC calcification that were representative of multiple pathways implicated in VC. Further studies are required to determine the precise roles of these genes in vitamin D sterol associated VC.
