# Supplementary material for: Chondro/Osteoblastic and Cardiovascular Gene Modulation in Human Artery Smooth Muscle Cells That Calcify in the Presence of Phosphate and Calcitriol or Paricalcitol
Source: J Cell Biochem. 2010 Jul 27;111(4):911–21. doi: 10.1002/jcb.22779 (PMC3470918; doi:10.1002/jcb.22779)
Supplement: Supplementary file 1 [file jcb0111-0911-SD1.doc]

| **Table 1. Genes that increase after exposure of human CASMC to vitamin D sterols** | | | | | | | | | |  |  |  |
| --- | --- | --- | --- | --- | --- | --- | --- | --- | --- | --- | --- | --- |
|  |  |  |  |  |  |  |  |  |  |  |  |  |
| **Gene ID** |  | **Gene name** | |  |  | **Time** | **BM** | **DM** | **Cal + DM** | **P value** | **Par + DM** | **P value** |
|  |  |  |  |  |  |  | **vs** | **vs** | **vs** |  | **vs** |  |
|  |  |  |  |  |  |  | **EM** | **BM** | **DM** |  | **DM** |  |
| ***Vitamin D metabolizing enzyme*** | | | |  |  |  |  |  |  |  |  |  |
| **CYP24A1** |  | Cytochrome P450, family 24, subfamily A, | | | | 4 hr |  |  | **3.99** | <0.0001 | **3.67** | <0.0001 |
| (-1365/-308) | | polypeptide 1 | |  |  | 1 d |  |  | **7.37** | <0.0001 | **4.76** | <0.0001 |
|  |  | 206504_at | |  |  | 3 d |  |  | **11.1** | <0.0001 | **5.52** | <0.0001 |
|  |  |  |  |  |  | 7 d |  |  | **10.1** | <0.0001 | **3.87** | 0.00059 |
| ***Chondro/osteoblast*** | | |  |  |  |  |  |  |  |  |  |  |
| **BMP-4 (BMP-2B)** | | Bone morphogenetic protein-4 (BMP-4) | | | | 1d | (-) | (+) | **1.05** | 0.03 | **1.03** | ns |
| (-236/-236) |  | 211518_s_at | |  |  | 3d | (-) |  | **1.2** | <0.0001 | **1.09** | 0.0125 |
|  |  |  |  |  |  | 7d | (-) | (-) | **1.33** | <0.0001 | **1.11** | 0.00013 |
| **BMP-6** |  | Bone morphogenetic protein-6 | | |  | 1d | (+) | (+) | **1.04** | 0.056 | **1.01** | ns |
| (-436/-148) |  | 206176_at | |  |  | 3d | (+) |  | **1.1** | <0.0001 | **1.04** | 0.0393 |
|  |  |  |  |  |  | 7d |  |  | **1.26** | <0.0001 | **1.11** | 0.00068 |
| **ALPL** |  | Alkaline phosphatase liver/bone/kidney | | | | 3d | (+) |  | **1.15** | 0.0004 | **1.11** | 0.004 |
| (-2399/-83) |  | 215783_s_at | |  |  | 7d |  |  | **1.06** | 0.006 | **1.06** | 0.01 |
| **TNFSF11** |  | Tumor necrosis factor (ligand) superfamily | | | | 1d |  |  | **1.17** | 0.00076 | **1.09** | 0.033 |
| (-1398) |  | member 11 (RANKL) | |  |  | 3d |  |  | **1.2** | 0.0015 | **1.03** | ns |
|  |  | 210643_at | |  |  |  |  |  |  |  |  |  |
| **TNFRSF11B** | | Tumor necrosis factor (receptor) superfamily | | | | 3d | (-) |  | **1.22** | 0.0026 | **1.22** | 0.0023 |
| (-2293/-595) | | member 11B, (Osteoprotegerin, OPG) | | | | 7d | (-) |  | **1.27** | <0.0001 | **1.25** | 0.00017 |
|  |  | 204932_at | |  |  |  |  |  |  |  |  |  |
| **TNFRSF11A** | | Tumor necrosis factor (receptor) superfamily | | | | 3d |  |  | **1.15** | 0.0007 | **1.099** | 0.011 |
| (-699/-169) |  | member 11A, NFKB activator (RANK) | | | | 7d |  |  | **1.12** | <0.00072 | **1.098** | 0.0033 |
|  |  | 238846_at | |  |  |  |  |  |  |  |  |  |
| **TGFB2** |  | Transforming growth factor beta 2 | | | | 1d |  |  | **1.58** | <0.0001 | **1.48** | <0.0001 |
| (-2162/-222) | | 209909_s_at | |  |  | 3d |  |  | **1.8** | <0.0001 | **1.45** | <0.0001 |
|  |  |  |  |  |  | 7d |  |  | **1.6** | <0.0001 | **1.35** | 0.00037 |
|  |  | 220407_s_at | |  |  | 1d |  |  | **1.41** | <0.0001 | **1.36** | <0.01 |
|  |  |  |  |  |  | 3d |  |  | **1.66** | <0.0001 | **1.38** | <0.01 |
|  |  |  |  |  |  | 7d |  |  | **1.3** | <0.0001 | **1.22** | <0.01 |
| **ANXA3** |  | Annexin A3 | |  |  | 7d |  |  | **1.39** | <0.0001 | **1.2** | 0.00048 |
| (-448/-129) |  | 209369_at | |  |  |  |  |  |  |  |  |  |
| **CILP** |  | Cartilage intermed layer protein nucleotide | | | | 1d |  |  | **1.13** | <0.0001 | **1.04** | ns |
| (-2036/-123) | | pyrophosphohydrolase | | |  | 3d |  |  | **1.5** | <0.0001 | **1.16** | 0.00026 |
|  |  | 206227_at | |  |  | 7d |  |  | **1.5** | <0.0001 | **1.15** | 0.00064 |
| **ATXN1** |  | Ataxin (Sca-1) | |  |  | 3d |  |  | **1.2** | <0.0001 | **1.09** | 0.016 |
| (-1440/-184) | | 203232_s_at | |  |  | 7d |  |  | **1.25** | 0.00002 | **1.08** | ns |
| **DKK1** |  | Dikkopf homolog 1 (Xenopus laevis) | | | | 3d | (+) | (+) | **1.12** | 0.001 | **1.05** | ns |
| (-279/-160) |  | 204602_at | |  |  | 7d |  |  | **1.39** | 0.01 | **1.35** | 0.018 |
| **HHIP** |  | Hedgehog interacting protein | | |  | 7d |  |  | **1.34** | <0.0001 | **1.23** | 0.0003 |
| (-129/-100) |  | 237466_s_at | |  |  |  |  |  |  |  |  |  |
| **DLX2** |  | distal-less homeo box 2 | | |  | 1d |  |  | **1.22** | 0.0007 | **1.1** | 0.05 |
| (-808/-258) |  | 207147_at | |  |  |  |  |  |  |  |  |  |
| **BAPX1** |  | bagpipe homeobox homolog 1 (Drosophila) | | | | 3d |  |  | **1.1** | 0.0003 | **1.05** | 0.026 |
| (-490/-141) |  | 207031_at | |  |  |  |  |  |  |  |  |  |
| ***Extracellular matrix*** | | |  |  |  |  |  |  |  |  |  |  |
| **ITGA8** |  | Integrin alpha 8 | |  |  | 1d |  |  | **1.13** | 0.0017 | **1.15** | 0.0006 |
| (-1173/-51) |  | 214265_at | |  |  | 3d | (-) |  | **1.36** | <0.0001 | **1.21** | 0.0001 |
|  |  |  |  |  |  | 7d | (-) |  | **1.4** | <0.0001 | **1.18** | 0.0013 |
| **TIMP1** |  | TIMP metallopeptidase inhibitor 1 | | | | 1d |  |  | **1.05** | 0.001 | **1.04** | 0.01 |
| (-1874/-183) | | 201666_at | |  |  | 3d |  |  | **1.09** | <0.0001 | **1.05** | 0.00012 |
|  |  |  |  |  |  | 7d | (-) |  | **1.07** | <0.0001 | **1.04** | 0.0004 |
| **COL4A6** |  | Collagen type IV, alpha 6 | | |  | 3d | (-) |  | **1.09** | 0.01 | **1.02** | ns |
| (-1274/-176) | | 213992_at | |  |  | 7d | (-) |  | **1.19** | <0.0001 | **1.11** | 0.04 |
| **COLXA1** |  | Collagen type X, alpha 1 | | |  | 7d | (+) |  | **1.08** | 0.005 | **1.05** | ns |
| (-2346/-40) |  | 205941_s_at | |  |  |  |  |  |  |  |  |  |
|  |  | 217428_s_at | |  |  | 7d | (+) |  | **1.1** | 0.05 | **1.02** | ns |
| **COL16A1** |  | Collagen type XVI, alpha 1 | | |  | 3d |  |  | **1.3** | <0.00001 | **1.11** | 0.009 |
| (-2302/-45) |  | 204345_at | |  |  | 7d | (+) |  | **1.33** | <0.0001 | **1.12** | 0.0024 |
| **COL22A1** |  | COLXXII, alpha 1 | |  |  | 1 | (-) |  | **1.24** | <0.00001 | **1.16** | <0.0001 |
| (-1134/-214) | | 228873_at | |  |  | 3 | (-) |  | **1.45** | <0.00001 | **1.24** | <0.00001 |
|  |  |  |  |  |  | 7 | (-) |  | **1.32** | <0.00001 | **1.21** | <0.0001 |
| ***Apoptosis*** | |  |  |  |  |  |  |  |  |  |  |  |
| **NLRP1** |  | NACHT, leucine rich repeat and PYD (pyrin | | | | 1d | (-) | (+) | **1.19** | <0.0001 | **1.06** | 0.0376 |
| (-2464/-158) | | domain) containing 1 | |  |  | 3d | (-) | (+) | **1.36** | <0.0001 | **1.16** | 0.0004 |
|  |  | 218380_at | |  |  | 7d | (-) |  | **1.35** | <0.0001 | **1.15** | 0.0046 |
| **PAWR** |  | PRKC, apoptosis, TW1, regulator | | | | 3d |  |  | **1.14** | 0.0025 | **1.11** | 0.0125 |
| (-763/-367) |  | 226231_at | |  |  | 7d |  |  | **1.2** | <0.0001 | **1.18** | 0.00036 |
| **GADD45B** | | Growth arrest and DNA damage inducible, | | | | 1d |  |  | **1.08** | 0.0129 | **1.03** | ns |
| (-900/-81) |  | beta |  |  |  | 3d |  |  | **1.16** | 0.00045 | **1.07** | 0.05 |
|  |  | 209304_x_at | |  |  | 7d | (+) |  | **1.17** | <0.0001 | **1.07** | 0.016 |
| ***Cell cycle/signal transduction*** | | | |  |  |  |  |  |  |  |  |  |
| **DUSP4** |  | Dual specificity phosphatase 4 | | |  | 7d | (-) | (+) | **1.16** | <0.0001 | **1.08** | 0.009 |
| (-1647/-24) |  | 204015_s_at | |  |  |  |  |  |  |  |  |  |
| **MAPK13** |  | mitogen activated protein kinase 13 | | | | 1d |  |  | **1.29** | <0.0001 | **1.16** | <0.001 |
| (-1757/-188) | | 210059_s_at | |  |  | 3d | (+) |  | **1.34** | <0.0001 | **1.13** | <0.001 |
|  |  |  |  |  |  | 7d | (+) |  | **1.21** | <0.0001 | **1.09** | <0.01 |
| **PTPRF** |  | protein tyrosine phosphatase, receptor | | | | 7d | (-) |  | **1.22** | <0.0001 | **1.11** | 0.01 |
| (-1079/-190) | | type F |  |  |  |  |  |  |  |  |  |  |
|  |  | 200636_s_at | |  |  |  |  |  |  |  |  |  |
| **SIRPA** |  | protein tyrosine phosphatase, non receptor | | | | 1d |  |  | **1.13** | 0.0002 | **1.1** | 0.0025 |
| (-1445/-58) |  | type substrate 1 | |  |  | 3d |  |  | **1.18** | <0.0001 | **1.09** | 0.007 |
|  |  | 202896_s_at | |  |  | 7d |  |  | **1.19** | <0.0001 | **1.12** | <0.0001 |
| **PLK2** |  | polo-like kinase 2 (Drosophila) | | |  | 1d |  |  | **1.2** | <0.01 | **1.11** | <0.05 |
| (-1882/-247) | | 201939_at | |  |  | 3d |  |  | **1.28** | <0.0001 | **1.16** | <0.01 |
|  |  |  |  |  |  | 7d |  |  | **1.18** | <0.0001 | **1.11** | <0.01 |
| ***Ion channels*** | |  |  |  |  |  |  |  |  |  |  |  |
| **SLC22A3** |  | solute carier family 22 | | |  | 1d |  |  | **1.46** | <0.0001 | **1.35** | <0.0001 |
| (-1441/-27) |  | 205421_at | |  |  | 3d |  |  | **2.14** | <0.0001 | **1.77** | <0.0001 |
|  |  |  |  |  |  | 7d |  |  | **1.94** | <0.0001 | **1.64** | <0.0001 |
| **KCNK3** |  | Potassium channel, subfamily K, member 3 | | | | 4h |  |  | **1.09** | 0.0173 | **1.13** | 0.00357 |
| (-977/-609) |  | 205952_at | |  |  | 1d |  |  | **1.43** | <0.0001 | **1.21** | <0.0001 |
|  |  |  |  |  |  | 3d | (-) |  | **1.47** | <0.0001 | **1.14** | <0.0001 |
|  |  |  |  |  |  | 7d | (-) |  | **1.49** | <0.0001 | **1.15** | 0.0017 |
| **VGCNL1** |  | Voltage gated channel like 1 | | |  | 7d | (-) |  | **1.2** | <0.0001 | **1.1** | 0.004 |
| (-490/-490) |  | 228608_at | |  |  |  |  |  |  |  |  |  |
| ***Other*** |  |  |  |  |  |  |  |  |  |  |  |  |
| **SOD3** |  | superoxide dismutase 3, extracellular | | | | 3d | (-) |  | **1.36** | <0.0001 | **1.2** | <0.0001 |
| (-125/-125) |  | 205236_x_at | |  |  | 7d | (-) |  | **1.33** | <0.0001 | **1.18** | 0.00078 |
| **DPP4** |  | Dipeptidylpeptidase 4(CD26,adenosine | | | | 1d |  |  | **1.18** | <0.0001 | **1.11** | 0.00287 |
| (-967/-306) |  | deaminase complexing p2) | | |  | 3d |  |  | **1.37** | <0.0001 | **1.2** | <0.0001 |
|  |  | 203717_at | |  |  | 7d |  | (-) | **1.42** | <0.0001 | **1.22** | <0.0001 |
| **HMCN1** |  | Hemicentin | |  |  | 1d | (-) | (+) | **1.09** | 0.027 | **1.06** | ns |
| (-1674/-123) | | 235944_at | |  |  | 3d |  |  | **1.26** | 0.0012 | **1.12** | ns |
|  |  |  |  |  |  | 7d |  |  | **1.37** | <0.0001 | **1.18** | 0.0046 |
| **RDH10** |  | Retinol dehydrogenase 10 (all-trans) | | | | 1d |  |  | **1.09** | 0.006 | **1.07** | 0.027 |
| (-2273/-223) | | 226021_at | |  |  | 3d |  |  | **1.2** | 0.00017 | **1.08** | 0.05 |
|  |  |  |  |  |  | 7d |  |  | **1.28** | <0.0001 | **1.11** | 0.00079 |
| **FZD7** |  | Frizzled homolog 7 (Drosophila) | | |  | 3d |  |  | **1.1** | 0.0095 | **1.05** | ns |
| (-2229/-153) | | 203705_s_at | |  |  | 7d |  |  | **1.14** | 0.003 | **1.1** | 0.02 |
| **IL6** |  | Interleukin 6 (interferon, beta 2) | | |  | 1d |  |  | **1.24** | <0.0001 | **1.19** | 0.00035 |
| (-1751/477) |  | 205207_at |  |  |  | 3d |  |  | **1.26** | <0.0001 | **1.14** | 0.0079 |
| **WNT2** |  | Wingless-type MMTV integration site family | | | | 7d |  | (-) | **1.16** | 0.00018 | **1.07** | 0.03 |
| (-2192/-172) | | member 2 |  |  |  |  |  |  |  |  |  |  |
|  |  | 205648_at | |  |  |  |  |  |  |  |  |  |
| **WNT16** |  | Wingless-type MMTV integration site family | | | | 1d |  |  | **1.16** | 0.037 | **1.17** | 0.027 |
| (-1215/-300) | | member 16 | |  |  |  |  |  |  |  |  |  |
|  |  | 224022_x_at | |  |  |  |  |  |  |  |  |  |
| **DCBLD1** |  | Discoidin,CUB and LCCL domain containing 1 | | | | 1d |  |  | **1.1** | 0.0095 | **1.05** | ns |
| (-927/-236) |  | 226609_at | |  |  | 3d | (+) |  | **1.21** | <0.0001 | **1.09** | 0.011 |
|  |  |  |  |  |  | 7d | (+) |  | **1.37** | <0.00001 | **1.19** | <0.0001 |
| **MEGF6** |  | EGF-like-domain multiple binding | | | | 3d | (-) |  | **1.1** | 0.0199 | **1.03** | ns |
| (-17/-17) |  | 213942_at | |  |  | 7d | (-) |  | **1.3** | <0.00001 | **1.13** | <0.0001 |
| **DSP** |  | Desmoplakin | |  |  | 1d | (-) | (+) | **1.15** | 0.0025 | **1.06** | ns |
| (-224/-224) |  | 200606_at | |  |  | 3d | (-) | (+) | **1.24** | <0.00001 | **1.16** | 0.0002 |
|  |  |  |  |  |  | 7d | (-) | (+) | **1.33** | <0.00001 | **1.21** | <0.0001 |
| **ARL4A** |  | ADP-ribosylation factor like 4 | | |  | 7d | (+) | (-) | **1.2** | <0.00001 | **1.08** | 0.014 |
| (-95/-95) |  | 205020_s_at | |  |  |  |  |  |  |  |  |  |
| **AXIN1** |  | Axin 1, wnt pathway | |  |  | 7d |  |  | **1.08** | 0.012 | **1.05** | ns |
| (-1319/-230) | | 212849_at | |  |  |  |  |  |  |  |  |  |
| **BCAT1** |  | branched chain aminotransferase 1, | | | | 1d | (-) |  | **1.14** | 0.0006 | **1.08** | 0.024 |
| (-1147/-543) | | cytosolic |  |  |  | 3d | (-) |  | **1.23** | <0.0001 | **1.11** | 0.013 |
|  |  | 225285_at |  |  |  | 7d | (-) |  | **1.35** | <0.0001 | **1.2** | 0.00018 |
| **PRR6** |  | proline rich 6 | |  |  | 1d |  |  | **1.08** | 0.021 | **1.06** | ns |
| (-497/-240) |  | 226611_s_at | |  |  | 3d | (+) |  | **1.16** | <0.001 | **1.1** | 0.0063 |
|  |  |  |  |  |  | 7d | (+) | (-) | **1.25** | <0.0001 | **1.18** | <0.0001 |
| **GCLC** |  | glutamate-cysteine ligase, catalytic subunit | | | | 4h |  |  | **1.15** | <0.001 | **1.12** | 0.0048 |
| (-2468/-459) | | 202923_s_at | |  |  | 1d |  |  | **1.23** | <0.0001 | **1.16** | <0.0001 |
|  |  |  |  |  |  | 3d |  |  | **1.36** | <0.0001 | **1.18** | <0.0001 |
|  |  |  |  |  |  | 7d |  |  | **1.34** | <0.0001 | **1.25** | <0.0001 |
| **FNDC4** |  | fibronectin type III, domain containing 4 | | | | 1d |  |  | **1.11** | 0.0023 | **1.08** | 0.019 |
| (-2078/-30) |  | 218843_at | |  |  | 3d | (+) |  | **1.18** | <0.0001 | **1.1** | 0.004 |
|  |  |  |  |  |  | 7d | (+) |  | **1.25** | <0.0001 | **1.11** | 0.0004 |
| **KRT14** |  | keratin 14 (epidermolysis bullosa simplex, | | | | 7d | (+) |  | **1.51** | <0.0001 | **1.16** | <0.05 |
| (-2286/-285) | | Dowling-Meara, Koebner | | |  |  |  |  |  |  |  |  |
|  |  | 209351_at | |  |  |  |  |  |  |  |  |  |
| **MFAP3L** |  | microfibrillar-associated protein 3-like | | | | 3d |  |  | **1.19** | 0.01 | **1.1** | <0.05 |
| (-2374/-380) | | 205442_at | |  |  | 7d | (-) |  | **1.45** | <0.0001 | **1.28** | <0.0001 |
| **SULT1E1** |  | sulfotransferase family 1E, estrogen- | | | | 3d | (-) |  | **1.35** | <0.0001 | **1.18** | <0.05 |
| (-86/-86) |  | preferring, member 1 | |  |  | 7d | (-) |  | **1.88** | <0.0001 | **1.36** | <0.001 |
|  |  | 222940_at | |  |  |  |  |  |  |  |  |  |
| **THBD** |  | Thrombomodulin | |  |  | 4h |  |  | **1.2** | **<0.05** | **1.2** | <0.05 |
| (-2388/-7) |  | 203887_s_at | |  |  | 1d |  |  | **1.4** | **<0.001** | **1.25** | <0.001 |
|  |  |  |  |  |  | 7d |  | (-) | **1.3** | **<0.001** | **1.1** | <0.001 |
|  |  |  |  |  |  |  |  |  |  |  |  |  |
| EM: DMEM/15%FBS | |  |  |  |  |  |  |  |  |  |  |  |
| BM: DMEM/15%FBS,ascorbic acid (50 ug/ml), dexamethasone (10-9 M) | | | | | |  |  |  |  |  |  |  |
| DM: differentiation medium (ascorbic acid, 50 ug/ml; dexamethasone, 10-9 M; beta-glycerolphosphate, 10 mM) | | | | | | | | | |  |  |  |
| FC: fold change | |  |  |  |  |  |  |  |  |  |  |  |
| AA: ascorbic acid; dex: dexamethasone; BGP: beta-glycerophosphate | | | | | |  |  |  |  |  |  |  |
| Cal: calcitriol: Par: paricalcitol | | |  |  |  |  |  |  |  |  |  |  |
| The affymetrix gene ID number is under the gene name | | | | |  |  |  |  |  |  |  |  |
| Some genes are members of more than one group | | | | |  |  |  |  |  |  |  |  |
| The number under the gene before the back slash is The VDRE site according to the highest predicted score; | | | | | | | | | |  |  |  |
| the number after the back slash is the VDRE site according to the closest site to the transcription start site | | | | | | | | | |  |  |  |
| (+), up-regulated genes comparing BM to expansion medium, EM; | | | | | |  |  |  |  |  |  |  |
|  | or DM to BM (p < 0.05) as designated in the column heading | | | | |  |  |  |  |  |  |  |
| (-), down-regulated genes comparing BM to expansion medium, EM; | | | | | |  |  |  |  |  |  |  |
|  | or DM to BM (p < 0.05) as designated in the column heading | | | | |  |  |  |  |  |  |  |
|  |  |  |  |  |  |  |  |  |  |  |  |  |
