# Supplementary material for: Chondro/Osteoblastic and Cardiovascular Gene Modulation in Human Artery Smooth Muscle Cells That Calcify in the Presence of Phosphate and Calcitriol or Paricalcitol
Source: J Cell Biochem. 2010 Jul 27;111(4):911–21. doi: 10.1002/jcb.22779 (PMC3470918; doi:10.1002/jcb.22779)
Supplement: Supplementary file 2 [file jcb0111-0911-SD2.doc]

| **Table 2. Genes that decrease after exposure of human CASMC to vitamin D sterols** | | | | | | | | | |  |  |  |
| --- | --- | --- | --- | --- | --- | --- | --- | --- | --- | --- | --- | --- |
|  |  |  |  |  |  |  |  |  |  |  |  |  |
| **Gene ID** |  | **Gene name** | |  |  | **Time** | **BM** | **DM** | **Cal + DM** | **P value** | **Par + DM** | **P value** |
|  |  |  |  |  |  |  | **vs** | **vs** | **vs** |  | **vs** |  |
|  |  |  |  |  |  |  | **EM** | **BM** | **DM** |  | **DM** |  |
| ***Mineralization inhibitor*** | | | |  |  |  |  |  |  |  |  |  |
| **ENPP1** |  | Ectonucleotide pyrophosphatase/ | | | | 3 d |  |  | **-1.13** | 0.002 | **-1.13** | 0.0025 |
| (-950/-15) |  | phosphodiesterase 1 | |  |  | 7 d |  |  | **-1.32** | <0.0001 | **-1.21** | <0.0001 |
|  |  | 205066_s_at | |  |  |  |  |  |  |  |  |  |
| **MGP** |  | Matrix gla protein | |  |  | 1d | (+) | (+) | **-1.1** | 0.028 | **-1.1** | 0.03 |
| (-1565/-149) | | 202291_s_at | |  |  |  |  |  |  |  |  |  |
| **IBSP** |  | Integrin-binding sialoprotein | | |  | 1d |  |  | **-1.18** | 0.01 | **-1.12** | ns |
| (-2058/-287) | | (bone sialoprotein II) | |  |  | 3d |  |  | **-1.35** | <0.0001 | **-1.3** | <0.0001 |
|  |  |  |  |  |  | 7d |  |  | **-1.24** | <0.0001 | **-1.16** | 0.0006 |
|  |  | 236028_at |  |  |  |  |  |  |  |  |  |  |
| ***Chondro/osteoblast*** | | |  |  |  |  |  |  |  |  |  |  |
| **WNT5A** |  | Wingless-type MMTV integration site family | | | | 1d |  |  | **-1.13** | 0.019 | **-1.09** | ns |
| (-1485/-151) | | member 5A | |  |  | 3d |  |  | **-1.34** | 0.0002 | **-1.26** | 0.0015 |
|  |  | 213425_at | |  |  | 7d |  |  | **-1.34** | 0.00019 | **-1.15** | 0.028 |
| **ASPN** |  | Asporin |  |  |  | 1d |  |  | **-1.2** | 0.001 | **-1.16** | 0.0051 |
| (-1727/-319) | | 219087_at | |  |  | 3d |  |  | **-1.36** | 0.00037 | **-1.28** | 0.0019 |
|  |  |  |  |  |  | 7d |  |  | **-1.24** | 0.03 | **-1.25** | 0.025 |
|  |  | 224396_s_at | |  |  | 7d |  |  | **-1.44** | 0.002 | **-1.27** | 0.024 |
| **DKK3** |  | Dikkopf homolog 3 (Xenopus Laevis) | | | | 3d |  |  | **-1.15** | 0.014 | **-1.14** | 0.021 |
| (-2337/-23) |  | 202196_s_at | |  |  | 7d |  |  | **-1.17** | 0.00029 | **-1.08** | 0.034 |
|  |  | 230508_at | |  |  | 1d |  |  | **-1.12** | 0.0026 | **-1.1** | 0.0087 |
|  |  |  |  |  |  | 3d |  |  | **-1.14** | 0.001 | **-1.11** | 0.008 |
|  |  |  |  |  |  | 7d |  |  | **-1.11** | 0.0006 | **-1.08** | 0.005 |
| **TGFBR2** |  | Transforming growth factor, beta receptor II | | | | 7d |  | (+) | **-1.2** | <0.0001 | **-1.1** | 0.0003 |
| (-1064/-496) | | 208944_at | |  |  |  |  |  |  |  |  |  |
| **CLEC3B** |  | C-type lectin domain family , member B | | | | 3d |  |  | **-1.22** | 0.0001 | **-1.18** | 0.00075 |
| (-1119/-488) | | 205200_at | |  |  | 7d |  |  | **-1.68** | <0.0001 | **-1.44** | 0.00014 |
| **POSTN** |  | Periostin, osteoblast specific factor | | | | 7d |  |  | **-1.15** | 0.0069 | **-1.17** | 0.0036 |
| (-191/-191) |  | 228481_at | |  |  |  |  |  |  |  |  |  |
| **SFRP2** |  | Secreted frizzled-related protein 2 | | | | 3d | (+) | (+) | **-1.08** | 0.01 | **-1.08** | 0.01 |
| (-1578/-313) | | 223122_s_at | |  |  | 7d | (+) |  | **-1.16** | 0.0085 | **-1.12** | 0.037 |
| **EDG2** |  | Endothelial diferentiation lysophosphatidic | | | | 1d |  |  | **-1.12** | 0.01 | **-1.13** | 0.005 |
| (-604/-96) |  | acid, GPCR2 | |  |  | 3d |  |  | **-1.12** | 0.003 | **-1.2** | 0.0024 |
|  |  | 204037_at |  |  |  | 7d |  |  | **-1.2** | <0.0001 | **-1.16** | <0.0001 |
| **TNC** |  | Tenascin C (hexabrachion) | | |  | 1d | (-) |  |  |  |  |  |
| (-1966/-84) |  | 201645_at | |  |  | 3d | (-) |  | **-1.19** | <0.001 | **-1.14** | 0.0089 |
|  |  |  |  |  |  | 7d | (-) |  | **-1.32** | <0.0001 | **-1.13** | 0.02 |
| **S100A4** |  | S100 calcium binding protein A4 (calcium | | | | 1d |  | (+) | **-1.2** | 0.00018 | **-1.2** | 0.00016 |
| (-1241/-575) | | protein, calvasculin) | |  |  | 3d | (+) | (+) | **-1.49** | <0.0001 | **-1.37** | <0.0001 |
|  |  | 203186_s_at | |  |  | 7d | (+) | (+) | **-1.33** | 0.00011 | **-1.26** | 0.00074 |
| **S100A16** |  | S100 calcium binding protein A16 | | | | 7d | (+) |  | **-1.09** | <0.01 | **-1.13** | <0.0001 |
| (-2396/-29) |  | 227998_at | |  |  |  |  |  |  |  |  |  |
| **FRZB** |  | Secreted frizzled-related protein 3 | | | | 7d |  |  | **-1.15** | 0.0076 | **-1.08** | ns |
| (-854/-425) |  | 203698_s_at | |  |  |  |  |  |  |  |  |  |
| ***Extracellular matrix*** | | |  |  |  |  |  |  |  |  |  |  |
| **COL5A3** |  | Collagen type 5, alpha 3 | | |  | 3d |  |  | **-1.19** | 0.01 | **1.16** | 0.027 |
| (-2379/-313) | | 218975_at | |  |  | 7d | (-) | (+) | **-1.15** | 0.008 | **1.1** | 0.05 |
|  |  | 52255_s_at | |  |  | 3d |  |  | **-1.21** | 0.013 | **-1.15** | 0.046 |
|  |  |  |  |  |  | 7d | (-) | (+) | **-1.15** | 0.014 | **-1.09** | ns |
| **COL11A1** |  | Collagen type XI, alpha 1 | | |  | 3d |  |  | **-1.07** | 0.045 | **-1.07** | 0.047 |
| (-561/-140) |  | 204320_at | |  |  |  |  |  |  |  |  |  |
| **MMP14** |  | Matrix metalloproteinase 14 | | |  | 7d |  |  | **-1.13** | 0.049 | **-1.16** | 0.02 |
| (-982/-121) |  | 217279_x_at | |  |  |  |  |  |  |  |  |  |
| **ADAMTS8** | | ADAM metallopeptidase with thrombo- | | | | 1d | (-) |  | **-1.08** | 0.011 | **-1.05** | 0.07 |
| (-345/-54) |  | spondin type 1 motif, 8 | | |  | 3d |  | (-) | **-1.17** | 0.0009 | **-1.11** | 0.014 |
|  |  | 235649_at | |  |  | 7d |  | (-) | **-1.19** | 0.00013 | **-1.16** | 0.0007 |
| **SPON2** |  | spondin 2, extracellular matrix protein | | | | 3d | (+) |  | **-1.1** | 0.007 | **-1.09** | 0.014 |
| (-1378/-65) |  | 218638_s_at | |  |  | 7d | (+) |  | **-1.31** | <0.0001 | **-1.28** | 0.0001 |
| ***Apoptosis*** | |  |  |  |  |  |  |  |  |  |  |  |
| **TNFRSF10D** | | Tumor necrosis factor receptor superfamily, | | | | 1d | (-) |  | **-1.11** | 0.0086 | **-1.1** | 0.009 |
| (-949/-70) |  | member 10d, decoy with truncated death | | | | 3d | (-) |  | **-1.15** | 0.00033 | **-1.16** | 0.0002 |
|  |  | domain |  |  |  |  |  |  |  |  |  |  |
|  |  | 227345_at | |  |  |  |  |  |  |  |  |  |
| **GIP3** |  | Interferon, alpha-inducible protein | | | | 3d | (+) |  | **-1.4** | 0.0075 | **-1.22** | ns |
| (-769/-221) |  | (clone IFI-6-16) | |  |  | 7d | (+) | (+) | **-1.82** | <0.0001 | **-1.65** | 0.00027 |
|  |  | 204415_at | |  |  |  |  |  |  |  |  |  |
| **BNIP3** |  | Bcl2/adenovirus E1B 19kDa interacting | | | | 3d | (-) |  | **-1.07** | 0.016 | **-1.08** | 0.012 |
| (-2491/-281) | | protein |  |  |  |  |  |  |  |  |  |  |
|  |  | 201849_at | |  |  |  |  |  |  |  |  |  |
|  |  |  |  |  |  |  |  |  |  |  |  |  |
| ***Cell cycle/signal transduction*** | | | |  |  |  |  |  |  |  |  |  |
| **PPAP2B** |  | Phosphatidic acid phosphatase type 2B | | | | 3d |  |  | **-1.16** | 0.0019 | **-1.14** | 0.0047 |
| (-378/-81) |  | 212226_s_at | |  |  | 7d |  |  | **-1.29** | <0.0001 | **-1.22** | <0.0001 |
| **PPF1A4** |  | Protein tyrosine phospahtase, receptor | | | | 7d | (-) |  | **-1.08** | <0.01 | **-1.07** | 0.01 |
| (-256/-153) |  | type, f polypeptide (PTPRF), interacting | | | |  |  |  |  |  |  |  |
|  |  | protein (liprin), alpha 4 | | |  |  |  |  |  |  |  |  |
|  |  | 214978_s_at | |  |  |  |  |  |  |  |  |  |
| **PGK1** |  | Phosphoglycerate kinase 1 | | |  | 7d | (-) |  | **-1.09** | <0.01 | **-1.1** | 0.026 |
| (-1363/242) |  | 200737_at | |  |  |  |  |  |  |  |  |  |
| ***Other*** |  |  |  |  |  |  |  |  |  |  |  |  |
| **HGF** |  | Hepatocyte growth factor (hepapoietin A; | | | | 3d |  |  | **-1.23** | 0.00085 | **-1.23** | 0.00097 |
| (-1868/-104) | | scatter factor | |  |  | 7d |  |  | **-1.77** | <0.0001 | **-1.44** | <0.0001 |
|  |  | 210997_at | |  |  |  |  |  |  |  |  |  |
| **NET02** |  | neurophilin (NRP) and tolloid (TLL)-like 2 | | | | 1d | (+) | (-) | **-1.18** | 0.0016 | **-1.2** | 0.016 |
| (-1770/-416) | | 222774_s_at | |  |  | 3d | (+) |  | **-1.49** | <0.0001 | **-1.36** | <0.0001 |
|  |  |  |  |  |  | 7d |  |  | **-1.84** | <0.0001 | **-1.47** | <0.0001 |
| **RBPSUH** |  | Recombining binding protein suppressor of | | | | 3d |  |  | **-1.14** | 0.0019 | **-1.12** | 0.0044 |
| (-1030) |  | hairless (Drosophila) | |  |  | 7d |  |  | **-1.18** | <0.0001 | **-1.11** | 0.0014 |
|  |  | 211974_x_at | |  |  |  |  |  |  |  |  |  |
| **TRIM16** |  | Tripartite motif-containing 16 similar to | | | | 7d | (-) |  | **-1.1** | 0.004 | **-1.13** | 0.0009 |
| (-1317/-29) |  | tripartite motif-containing 16; | | |  |  |  |  |  |  |  |  |
|  |  | estrogen-responsive B box protein | | | |  |  |  |  |  |  |  |
|  |  | 204341_at | |  |  |  |  |  |  |  |  |  |
| **CA12** |  | Carbonic anhydrase XII | | |  | 3d |  |  | **-1.11** | 0.008 | **-1.12** | 0.0049 |
| (-1292/-70) |  | 203963_at | |  |  | 7d |  |  | **-1.35** | <0.00001 | **-1.24** | <0.0001 |
|  |  | 204508_s_at | |  |  | 3d |  |  | **-1.12** | 0.017 | **-1.15** | 0.006 |
|  |  |  |  |  |  | 7d | (-) |  | **-1.41** | <0.00001 | **-1.3** | <0.0001 |
| **SMAP1L** |  | Stromal membrane-associated protein 1-like | | | | 7d | (-) |  | **-1.16** | <0.0001 | **-1.14** | <0.0001 |
| (-1755/-24) |  | 225282_at | |  |  |  |  |  |  |  |  |  |
| **THBS1** |  | Thrombospondin | |  |  | 7d |  |  | **-1.6** | <0.001 | **-1.1** | n.s. |
| (-1982/-219 |  | 201107_s_at | |  |  |  |  |  |  |  |  |  |
| ***Smooth muscle cell contraction*** | | | |  |  |  |  |  |  |  |  |  |
| **CAV1** |  | caveolin 1 |  |  |  | 3d |  |  | **-1.11** | 0.00016 | **-1.09** | 0.00077 |
| (-1762/-518) | | 203065_s_at | |  |  | 7d |  |  | **-1.23** | <0.00001 | **-1.14** | 0.0001 |
| **CORIN** |  | Corin, serine peptidase | | |  | 3 | (+) |  | **-1.17** | <0.0001 | **-1.08** | 0.008 |
| (-38/-38) |  | 220356_at | |  |  | 7 | (+) | (+) | **-1.2** | 0.0001 | **-1.15** | 0.0016 |
|  |  | 239260_at | |  |  | 1d |  | (+) | **-1.09** | 0.012 | **-1.08** | 0.028 |
|  |  |  |  |  |  | 3 | (+) |  | **-1.17** | 0.0011 | **-1.11** | 0.019 |
|  |  |  |  |  |  | 7 | (+) | (+) | **-1.25** | 0.0002 | **-1.18** | 0.0026 |
|  |  |  |  |  |  |  |  |  |  |  |  |  |
| EM: DMEM/15%FBS | | |  |  |  |  |  |  |  |  |  |  |
| BM: DMEM/15%FBS,ascorbic acid (50 ug/ml), dexamethasone (10-9 M) | | | | | |  |  |  |  |  |  |  |
| DM: differentiation medium (ascorbic acid, 50 ug/ml; dexamethasone, 10-9 M; beta-glycerolphosphate, 10 mM) | | | | | | | | | |  |  |  |
| FC: fold change | |  |  |  |  |  |  |  |  |  |  |  |
| AA: ascorbic acid; dex: dexamethasone; BGP: beta-glycerophosphate | | | | | |  |  |  |  |  |  |  |
| Cal: calcitriol: Par: paricalcitol | | |  |  |  |  |  |  |  |  |  |  |
| The affymetrix gene ID number is under the gene name | | | | |  |  |  |  |  |  |  |  |
| Some genes are members of more than one group | | | | |  |  |  |  |  |  |  |  |
| The number under the gene before the back slash is The VDRE site according to the highest predicted score; | | | | | | | | | |  |  |  |
| the number after the back slash is the VDRE site according to the closest site to the transcription start site | | | | | | | | | |  |  |  |
| (+), up-regulated genes comparing BM to expansion medium, EM; | | | | | |  |  |  |  |  |  |  |
|  | or DM to BM (p < 0.05) as designated in the column heading | | | | | |  |  |  |  |  |  |
| (-), down-regulated genes comparing BM to expansion medium, EM; | | | | | |  |  |  |  |  |  |  |
|  | or DM to BM (p < 0.05) as designated in the column heading | | | | | |  |  |  |  |  |  |
