# Supplementary material for: Chondro/Osteoblastic and Cardiovascular Gene Modulation in Human Artery Smooth Muscle Cells That Calcify in the Presence of Phosphate and Calcitriol or Paricalcitol
Source: J Cell Biochem. 2010 Jul 27;111(4):911–21. doi: 10.1002/jcb.22779 (PMC3470918; doi:10.1002/jcb.22779)
Supplement: Supplementary file 3 [file jcb0111-0911-SD3.doc]

| **Table 3.** | **Branched DNA (bDNA) analysis confirms human CASMC gene expession changes after** | | | | | | | | | |  |
| --- | --- | --- | --- | --- | --- | --- | --- | --- | --- | --- | --- |
|  | **7 days of exposure to vitamin D sterols** | | | | | | |  |  |  |  |
|  |  |  | |  | | |  |  |  |  |  |
|  |  |  | |  | | |  |  |  |  |  |
|  | **Gene** |  | |  | | | **BM** | **DM** | **DM** | **Cal + DM** | **Par + DM** |
|  |  |  | |  | | | **vs** | **vs** | **vs** | **vs** | **vs** |
|  |  |  | |  | | | **EM** | **EM** | **BM** | **DM** | **DM** |
|  |  |  | |  | | |  |  |  |  |  |
| ALP | Alkaline phosphatase | | | | 1.13 | | | 1.00 | -1.13 | **1.47** | 1.31 |
|  |  |  | |  | | |  |  |  |  |  |
| MGP | Matrix gla protein | |  | | | 1.33 | | **1.60** | 1.20 | 1.08 | -1.10 |
|  |  |  | |  | | |  |  |  |  |  |
| FBN1 | Fibrillin 1 |  | |  | | | -1.16 | -1.09 | 1.06 | 1.04 | 1.08 |
|  |  |  | |  | | |  |  |  |  |  |
| OPG | Osteoprotegerin | |  | | | **-2.38** | | **-2.11** | 1.13 | **2.13** | **1.82** |
|  |  |  | |  | | |  |  |  |  |  |
| FRZB (SFRP3) | Secreted frizzled related protein-3 | | | | 1.61 | | | **1.95** | 1.20 | -1.25 | -1.08 |
|  |  |  | |  | | |  |  |  |  |  |
| ENPP1 | Ectonucleotide pyrophosphatase/ | | | | -1.09 | | | -1.13 | -1.03 | **-1.52** | -1.40 |
|  | phosphodiesterase 1 | |  | | |  | |  |  |  |  |
|  |  |  | |  | | |  |  |  |  |  |
| RUNX2 | Runt related transcription factor 2 | | | | 1.37 | | | 1.37 | -1.00 | 1.04 | -1.09 |
|  |  |  | |  | | |  |  |  |  |  |
| EM: DMEM/15% fetal bovine serum | | |  | | |  | |  |  |  |  |
| BM: DMEM/15% FBS, ascorbic acid (50 ug/ml), dexamethasone (10-9 M) | | | | | | | |  |  |  |  |
| DM: DMEM/15%FBS,aa,dex,beta-glycerol phosphate (10 mM) | | | | | | | |  |  |  |  |
| The data were normalized to cyclophin levels and then were transformed using log2 | | | | | | | | |  |  |  |
| The values displayed here are fold change (or negative reciprocal if fc < 1) of the treatment 1 - treatment 2, where treatment 1 and 2 are given as column names | | | | | | | | | | | |
| Bold values: Fold changes are not equal to 1 (p<0.05) | | | | | | | |  |  |  |  |
